# Cross-Cultural Adaptation of the Greek Versions of the Lysholm Knee Scoring Scale and Tegner Activity Scale

**DOI:** 10.7759/cureus.9372

**Published:** 2020-07-24

**Authors:** Andreas Panagopoulos, Evdokia Billis, Georgios-Rafail Floros, Theodoros Stavropoulos, Evangelia Kaparounaki, Markela Moucho, Athanasia Paskou, Yelverton Tegner

**Affiliations:** 1 Orthopaedics, University of Patras Medical School, Patras, GRC; 2 Physiotherapy, University of Patras School of Health Rehabilitation Sciences, Patras, GRC; 3 Orthopaedics, University of Patras, Patras, GRC; 4 Orthopaedics, Universtity of Patras, Patras, GRC; 5 Health Sciences, Luleå University of Technology, Luleå, SWE

**Keywords:** outcome assessment, questionnaire, validity, translation, greek language, cultural adaptation, lysholm score, tegner activity scale

## Abstract

Purpose

The Lysholm Knee Scoring Scale (LKSS) and the Tegner Activity Scale (TAS) are widely used instruments for assessing knee function and activity level in various knee pathologies, especially knee ligament injuries. The purpose of this study was to translate and cross-culturally adapt the Greek versions of the Lysholm Knee Scoring Scale (Gr-LKSS) and Tegner Activity Scale (Gr-TAS) and assess their reliability and validity in Greek patients suffering from various knee problems.

Materials and methods

Translation of the LKSS and TAS questionnaires was done according to established international guidelines. Fifty-five patients (32 males and 23 females; mean age: 24 ±7 years; range: 17-54 years) with various knee pathologies completed the Gr-LKSS and Gr-TAS along with the Greek versions of International Knee Documentation Committee (IKDC) Subjective Knee Evaluation Form, the Knee Outcome Survey Activities of Daily Living Scale (KOS-ADLS) and the Oxford Knee Score (OKS). Test-retest reliability was evaluated with the intraclass correlation coefficient (ICC) in 53 (96%) individuals, who completed the questionnaires again after 48-72 hours while abstaining from all forms of treatment. Internal consistency for the Gr-LKSS was measured using Cronbach’s alpha and criterion-related validity was evaluated with the Pearson's correlation coefficient (r) in relation to control questionnaires (IKDC, KOS-ADLS, OKS). The distribution of floor and ceiling effects were also determined.

Results

There were no problems during the forward-backward translation and cultural adaptation of the Gr-LKSS and Gr-TAS. Criterion-related validity was confirmed with moderate to high associations of Gr-LKSS and Gr-TAS (after injury) with the IKDC and KOS-ADLS (Pearson’s r ranging between 0.61-0.71 and 0.64-0.73, respectively). However, weak correlations were yielded between both questionnaires with the OKS (r=0.14-0.19). The internal consistency for Gr-LKSS was high (Cronbach's alpha: 0.779) and the test-retest reliability was high for both questionnaires (Gr-LKSS: ICC=0.950; Gr-TAS before and after injury: ICCs of 0.877 and 0.876, respectively).

Conclusion

The cultural adaptation and validity of Gr-LKSS and Gr-TAS were successfully accomplished. These questionnaires are recommended for use in the evaluation of soft tissue knee disorders in both clinical practice and research.

## Introduction

Knee injuries represent a serious public health burden, given their high incidence across the age continuum and the frequent need for surgical treatment and long-term rehabilitation. Gage et al. evaluated 6,664,324 knee injuries presented to the US emergency departments from 1999 through 2008 and reported a rate of 2.29 knee injuries per 1,000 population, with those between 15-24 years of age having the highest injury rate (3.83), and with strains or sprains (42.1%) being the most common diagnoses [1]. It is imperative to use a validated tool to evaluate the injury status and clinical outcome of these patients irrespective of subsequent treatment change. Knee joint-specific measures or scales are designed to focus on clinical manifestations and on the patients' subjective symptoms to evaluate the impact of the injury on knee function and overall quality of life, which may offer better diagnosis, more precise picture of knee status, and possible treatment options. Many outcome measures are applicable to all knee problems with variable sensitivity [2]. In general, outcome measures need to have high validity and reliability, namely measuring what they are supposed to and showing the least possible test-retest variability respectively. They should also be adequately responsive by being sensitive to change over time [3].

The Lysholm Knee Scoring Scale (LKSS) and Tegner Activity Scale (TAS) are two popular patient-reported instruments that measure outcomes in patients with knee problems [4]. The LKSS score was developed in 1982 to determine patients' functional status after anterior cruciate ligament (ACL) reconstruction. It was further revamped and refined to include only subjective items in 1985 [5]. The TAS was developed as an additional instrument meant to complement the LKSS and designed to assess activity level based on work and sports activities. Both scores have been used in numerous publications worldwide, and their reliability, validity, and responsiveness have been established for various knee problems, including ACL and meniscal injuries [3,6,7], patellofemoral pain syndrome [8], medial patellar plica syndrome [9], patellar dislocation [10], various chondral disorders [11], knee arthroplasty [12], as well as in individuals with normal knees [13]. So far, both questionnaires have been translated, validated, and culturally adapted into several different languages, and they have all shown good internal consistency, test-retest reliability, and external validity [6,8,11-21].

Although informally translated versions of both the LKSS and TAS have been used in Greek Orthopaedic Society, officially translated and culturally adapted versions have not yet been provided. Cross-cultural adaptations may contribute to a better understanding of the measurement properties of the questionnaires. The need for validated translations has become essential with the growing number of multicenter and multinational studies, which provide more statistical power to randomized controlled trials. Given the prevalence and socioeconomic impact of knee disorders, we believe that a Greek cultural adaptation and validation procedure of the LKSS and TAS would be extremely beneficial for Greek-speaking surgeons, allied health professionals, and patients.

Hence, the purpose of the present study was to cross-culturally adapt the LKSS and TAS into the Greek language (Gr-LKSS and Gr-TAS) and setting and to evaluate their reliability and validity in patients with various knee disorders.

## Materials and methods

A. Development of usable test documents (all five scoring methods)

This initial workgroup focused its efforts on creating brief and straightforward test documents without affecting the primary scores and the guidelines' overall quality and validity. Their ultimate goal was to formulate every single test in a way that would fit in an A4-sized page, adding to the score’s flexibility in terms of an easy-filling and storage-friendly form. It is known that in clinical practice, larger, multi-paged questionnaires are usually quite cumbersome as individual parts may be lost or left blank by the patient. This patient folder included a front page with the patient’s demographic data, injury, and treatment details together with five different pages, each containing one of the scoring systems that were to be examined: Gr-LKSS, Gr-TAS, International Knee Documentation Committee (IKDC) Subjective Knee Evaluation Form, the Knee Outcome Survey Activities of Daily Living Scale (KOS-ADLS), and Oxford Knee Score (OKS).

B. Translation procedure of Gr-LKSS and Gr-TAS

Translation and cross-cultural adaptation of the reference English LKSS and TAS into Greek was performed in five stages, consistent with the stages recommended by Guillemin [22] and the principles of the Professional Society for Health Economics and Outcomes Research (ISPOR) Task Force guidelines for translation and cultural adaptation of patient-reported outcomes.

Four individual conversions were conducted, translating the initial English scores into Greek. All participants in this process were native Greek speakers and they completed the forward translations independently. Two were conducted by associate professors, one in orthopedics (AP) and one in physiotherapy (EB), with excellent and certified knowledge of English and prior experience of involvement in other similar projects. The other two conversions were performed by a certified independent professional translator and a bilingual fifth-year medical student (GF). Results of the four individual conversions were gathered and evaluated by the project coordinator (AP), and a primary edition of Gr-LKSS and Gr-TAS was assembled through cross-editing of the four individual translations. During this procedure, there were no significant differences observed between the four individual translations and the initial English form. Backward translation was performed by three individual translators, who were not involved in any of the previous steps. Two of them were orthopedic residents in our clinic with excellent and certified knowledge of English, and the other one was a native English professional translator with no prior knowledge of the LKSS. A back-translation check was carried out by the project supervisor (AP) in collaboration with the other authors. Back-translated texts were compared to the text-sources (Greek language), checking for any misinterpretations or mistakes. No significant inconsistencies were noted during this procedure, thus establishing an equivalence in meaning for our questionnaires in relation to the sources. All back-translated questionnaires were compared to the initial English scores by the project’s supervisor and the two independent official translators of each previous step (forward and backward translation). In the final step, all researchers and translators convened and had discussions to solve any discrepancies and other language expression issues that existed in the questionnaire. Thus, the pre-final versions of Gr-LKSS and Gr-TAS were obtained.

Testing of the pre-final Greek versions of the questionnaires in order to determine the acceptability and comprehensibility of their translations was done on 11 patients at our clinic during their preoperative assessment for undergoing scheduled ACL reconstructive surgery. All patients were native Greek speakers and were diagnosed with an ACL injury. For every single item of each questionnaire, patients were asked to answer the following questions: “do you understand the meaning of this?’’ and “can you describe the meaning of this in your own words?’’ in order to evaluate the clarity of the score’s questions. Two independent residents of our clinic, not involved in the whole procedure, administered and collected the forms. The project’s supervisor assessed these preliminary results, and the necessary alterations and improvements were made leading to the creation of the final version of the questionnaires. In the final step of this procedure, the finalized text of the Greek version of LKSS and TAS was delivered to an officially certified proficient user of Greek language for syntax and grammar check (Appendices 1 and 2).

C. Outcome tools

The LKSS was first published in 1982 [4] and revised in 1985 [5]. The score ranges from 0 to 100 points and is divided into eight sections that assess instability (25 points), pain (25 points), catching (15 points), stair-climbing (10 points), swelling (10 points), and need for support, squatting, and limping (5 points each). Scores are categorized as excellent (95-100), good (84-94), fair (65-83), and poor (≤64). Original and revised scores were intended for in-person clinician administration, although subsequent studies have documented using the score as a patient-completed questionnaire [2,10]. There are consistent reports of no floor or ceiling effects (i.e., <15% of patients scoring the lowest or highest score, respectively) [3,5,8,9]. Ra et al. have reported no significant differences between the IKDC subjective and the LKSS in terms of the amount of ceiling effect and the correlation with one leg hope scale in patients with ACL reconstruction [23]. However, the ceiling effect of the Lysholm score was greater than the IKDC subjective score (30.6% vs 17.2% respectively at 12 months postoperatively), and this should be addressed when assessing the patient's functional status postoperatively.

The TAS is a one-item instrument developed to complement the LKSS, based on observations that limitations in function scores (Lysholm) may be masked by a decrease in activity level [3,5]. It evaluates the patient’s level of work and sports activity on an 11-level scale, with higher scores representing higher levels of physical activity. A score of 0 represents “sick leave or disability pension because of knee problems”, whereas a score of 10 corresponds to participation in elite competitive sports. Activity levels of 6-10 can only be achieved if the person participates in recreational or competitive sport. Studies have consistently reported no floor or ceiling effects in those with a knee injury or osteoarthritis (OA) [3,5,8,12].

The IKDC score was introduced in 1987 to develop a standardized international documentation system for knee conditions. The IKDC Standard Knee Evaluation Form, which was designed for knee ligament injuries, was subsequently published in 1993 and revised in 1994 [24]. The IKDC Subjective Knee Evaluation Form was developed as a revision of the Standard Knee Evaluation Form in 1997. It has undergone subsequent minor revisions since its publication in 2001 [25]. IKDC is the standard form for use in all publications on results of treatment of knee ligament and chondral injuries and is supported by several international committees [International Cartilage Regeneration and Joint Preservation Society (ICRS); The American Orthopaedic Society for Sports Medicine (AOSSM); European Society of Sports Traumatology, Knee Surgery and Arthroscopy (ESSKA), and Asia Pacific Orthopaedic Society for Sports Medicine (APOSSM)]. It is a reliable and valid knee-specific measure of symptoms, function, and sports activity, which is appropriate for patients with a wide variety of knee problems. The subjective IKDC form consists of 18 sections divided into three categories: (a) symptoms, including pain, stiffness, swelling, locking/catching, and giving way; (b) sports and daily activities; and (c) current knee function and knee function prior to knee injury (not included in the total score). Scores for each item are summed up to give a total score (excluding item 10a). The total score is calculated as (sum of items)/(maximum possible score) x 100, to give a total score of 100. The IKDC was validated for Greek patients with knee-related injuries in 2016 [26].

The OKS is a 12-item patient-reported outcome score, specifically designed and developed to assess function and pain after total knee replacement (TKR) surgery. It involves a single index pertaining to knee pain and function (pain severity, mobility, limping, stairs, standing after sitting, kneeling, giving way, sleep, personal hygiene, housework, shopping, and transport). Each item is followed by five responses (scores ranging from 1-5, where 1 = best and 5 = worst outcomes) regarding the function of the knee in the previous four weeks. In the original version, the total score ranged from 12-60, where higher scores reflected poor outcomes and lower scores reflected better outcomes. It is short, reproducible, valid, and sensitive to clinically important changes. In a recent study by Harris et al. based on the National Health Service Patient Reported Outcome Measures (NHS PROMs) data, the OKS did not exhibit a ceiling or floor effect overall, or for both its pain and function subscales and remains a valid measure of outcomes for patients undergoing total knee arthroplasty (TKA) [27]. The OKS was translated and validated for Greek patients with knee OA in 2015 [28].

The KOS-ADLS is a patient-reported instrument designed to measure functional status relating to knee pathology and impairment [29]. It consists of two separate scales, the Activities of Daily Living Scale (ADLS), which is designed to measure symptoms and functional limitations during activities of daily living, and the Sports Activity Scale (SAS), which is intended to measure symptoms and functional limitations during sports activities. The ADLS consists of 14 items (items 1-6 measure symptoms commonly experienced during daily living activities, whereas items 7-14 are related to functions during activities of daily living). Responses for each item are scored from 0-5, with the exception of item 9 (0-3) and item 10 (0-2), and the total score is calculated as the sum of scores from the responses to each item; it is then transformed to a percentage score by dividing by the maximum total possible score and multiplying by 100. Acceptable ceiling effects have been reported in people with a variety of knee pathologies undergoing physical therapy and orthopedic surgeon evaluation [29]. The KOS-ADSL was translated and validated for Greek patients with a variety of pathological knee disorders in 2011 [30].

D. Sample

Power analysis showed that a minimum of 50 patients was required for floor or ceiling effects, reliability, and validity analyses. Fifty-five consecutive patients (32 males and 23 females; mean age: 23.5 years; range 17-54 years) with various knee pathologies (Table 1) were seen at the orthopedic outpatient clinic of our university between January and March 2020. Exclusion criteria were any kind of inflammatory or posttraumatic knee arthritis, infectious disease, age of <16 years, poor knowledge of Greek language, and the inability to understand and read Greek texts. Patient sex, age, medical history, injury history, educational status, occupation, diagnosis (including advanced imaging), and type of intervention (conservative or surgical) were recorded (Table 1). This study was approved by the ethical committee of Patras University Hospital (IRB number: 456/15.1.2020), and all patients gave their informed consent upon receiving complete information on the study and agreed to the presented protocol.

E. Data analysis

To evaluate the feasibility of the questionnaires, we asked each patient if they had any difficulty understanding the content. We calculated the miss rate of every item, and a >5% miss rate of a certain item suggested an existing problem regarding acceptability. We also recorded the average time (in minutes) required to complete the questionnaires.

Internal consistency represents the correlation between items that build up the score and is assessed using Cronbach’s alpha coefficient with a 95% confidence interval (CI). This parameter represents the degree to which every test item proposed to assess knee function produces similar scores. According to the literature, a test sample of 30-40 patients and an alpha value from 0.78 to 0.97 indicates good homogeneity [15,17,20].

Test-retest reliability represents the degree to which subsequent test measurements, under the same circumstances, produce consistent results over time and was investigated by calculating the intraclass correlation coefficient (ICC, two-way random model for agreement) between test and re-test measurements. The interclass correlation coefficient ranges between 0.00 and 1.00. Values in the region of 0.75 to 1.00 show excellent internal correlation. To assess the consistency and reliability of the procedure, 53/55 of these patients were re-evaluated again after completing the reference tests (Gr-LKSS and Gr-TAS), 48-72 hours after the initial test, without having received any form of treatment in the meantime.

Criterion-related validity examines whether a test “measures what it claims’’ and is evaluated in relation to other tests already validated to serve the same purpose. The Gr-LKSS and Gr-TAS were correlated with the Greek versions of IKDC, KOS-ADLS, and OKS using Pearson’s correlation coefficient (r). According to the literature, a score has adequate construct validity when Pearson’s r reaches rates close to 0.8.

Floor and ceiling effects were also calculated and were considered to be present if more than 15% of the respondents achieved the lowest or highest possible total score [8]. In addition to the analysis of the Gr-LKSS and Gr-TAS total scores, the single items of the Gr-LKSS were analyzed identically for any floor or ceiling effects. All results were statistically analyzed with the SPSS Statistics for Windows version 25 (IBM, Armonk, NY). Data were reported as mean, standard deviations, frequencies, and percentages.

## Results

Cross-cultural translation process

The translation of the LKSS and TAS from English into Greek was performed without any difficulties and with approval and contribution from YT, the senior author of the original publication of the questionnaires. No major inconsistencies were found regarding forward and backward translation procedures, as both questionnaires did not include any elements that could vary significantly among different cultures and lifestyles (daily personal hygiene, eating manners, etc.). In the TAS, “hockey” was omitted as this sport is not at all popular in Greece.

Feasibility

The two questionnaires (Gr-LKSS and Gr-TAS) were easily completed by all patients, and no significant missing responses, gender differences, and other language difficulties were noted for any specific question. The average time required to complete was 5.8 minutes for Gr-LKSS and 3.5 minutes for Gr-TAS. All patients found the questions relevant to their health status and physical activities.

Internal consistency

Cronbach’s alpha yielded excellent values for LKSS, both at baseline (a=0.779) and during the repeated measurement (a=0.776), indicating very good internal consistency of the measure. TAS, being a one-item questionnaire, was not considered for internal consistency testing.

Validity testing

Gr-LKSS yielded strong associations and statistically significant results with KOS-ADSL (r=0.713) and IKDC (r=0.735) questionnaires, indicating good criterion-related validity. However, LKSS demonstrated weak associations with OKS (r=0.197). Gr-TAS before-injury also yielded poor associations with all three questionnaires (KOS: r=0.064; OKS-ADSL: r=-0.166, and IKDC: r=0.061). Values of TAS after injury had better associations with KOS-ADLS (r=0.635) and IKDC (r=0.675), but still showed a weak correlation with OKS (r=-0.138). Pearson’s correlations between LKSS and TAS questionnaires are summarized in Table 2.

Test-retest reliability

Fifty-three subjects were re-evaluated after completing the reference tests, 48-72 hours after the initial test (we lost track of two subjects). ICC estimates and their 95% CIs were calculated for both questionnaires as well as the standard error of measurement (SEM) and smallest detectable difference (SDD) values (Table 3). The test-retest reliability for the Gr-LKSS was high (ICC=0.950, SEM=3.36, SDD=12.48%), as was for the Gr-TAS before injury (ICC=0.877, SEM=0.66, SDD=24.79%) and after injury (ICC=0.876, SEM=0.96, SDD=62.55%).

Floor-ceiling effects

No floor or ceiling effects (worst and best score, respectively) were detected across LKSS, as there were no scores between either 0-5% (floor) or between 95-100% (ceiling effect). TAS also reported no floor effects. Although 14.5% scored the highest score (10 in pre-injury retest administration of TAS), it is the nature of this single-item questionnaire that allows this high scoring, representing the maximum physical activity level of the patient. The distribution of floor and ceiling effects of each item’s questionnaire is illustrated in Table 4.

**Table 1:** Demographic and clinical profile of the study sample (n=55) SD: standard deviation; KOS: Knee Outcome Survey; OKS: Oxford Knee Score; IKDC: International Knee Documentation Committee; ACL: anterior cruciate ligament; MRI: magnetic resonance imaging; PLC: posterolateral corner; PCL: posterior cruciate ligament

Characteristics	Minimum–Maximum	Mean (SD)
Age	17–54	24 (7)
Height (cm)	1.57–1.95	1.76 (0.09)
Weight (kg)	48–95	70.41 (11.13)
Lysholm	39–95	74.69 (14.9)
Tegner: before injury	3–10	7.35 (1.9)
Tegner: after injury	0–10	4.33 (2.7)
KOS	20.00–97.14	77.84 (18.35)
OKS	20.80–93.7	53.37 (18.16)
IKDC	14.9–97.7	66.03 (17.63)
Second measurements		
Lysholm	41–95	75.06 (15.04)
Tegner: before injury	3–10	7.36 (1.86)
Tegner: after injury	0–10	4.21 (2.7)
		Frequency (Percent)
Education		
Secondary / university		7 (13%) / 48 (87%)
Dominant leg		
Right / left		40 (73%) / 15 (27%)
Diagnosis		
ACL rupture		10 (18%)
Combined ACL injury (meniscal tear, tendon, etc.)		7 (13%)
Isolated meniscal lesion (lateral 5, medial 5, horizontal 6, radial 1, complex 1, bucket-handle 2)		10 (18%)
Medial collateral ligamentous injury		2 (4%)
Patellofemoral (subluxation, chondromalacia, etc.)		8 (15%)
Patella tendon (2 tendinitides, 1 after reconstruction for acute rupture)		3 (5%)
Multiple knee injury lesion (5 menisci and cartilage lesion, 5 ACL and PLC injury, 4 ACL and PCL injury, 1 knee dislocation ACL-PCL-PLC)		15 (27%)
MRI		26 (47%)
Surgical treatment		19 (34%)
Conservative treatment		36 (65%)

**Table 2:** Association of Lysholm and Tegner Activity Scales with other scores (n=55) *Correlation (Pearson’s) is significant at 0.01 level (two-tailed) KOS-ADLS: Knee Outcome Survey Activities of Daily Living Scale; OKS: Oxford Knee Score; IKDC: International Knee Documentation Committee

Questionnaire	Lysholm Score	Tegner: Before Injury	Tegner: After Injury
KOS-ADLS	0.713^* ^(p<0.001)	0.064 (p=0.642)	0.635^* ^(p<0.001)
OKS	0.197 (p=0.149)	-0.166 (p=0.227)	-0.138 (p=0.317)
IKDC	0.735^* ^(p<0.001)	0.061 (p=0.66)	0.675^* ^(p<0.001)

**Table 3:** Test-retest reliability results (n=53) ICC: intraclass correlation coefficient; SEM: standard error of measurement; SDD: smallest detectable difference

	ICC	95% Confidence Interval (Lower Bound–Upper Bound)	SEM	SDD
Lysholm score	0.95	0.914–0.971	3.36	12.48%
Tegner: before injury	0.87	0.796–0.927	0.66	24.79%
Tegner: after injury	0.87	0.795–0.927	0.96	62.55%

**Table 4:** Distribution, floor, and ceiling effects of each item within the Greek Lysholm and Tegner scales (n=55) SD: standard deviation

	Mean (SD)	Observed Range	Reported Range	Floor Effect Percent (%)	Ceiling Effect Percent (%)
Lysholm Knee Score					
Limp (Q1)	1.55 (0.6)	0-5	1-3	5.5	50.9
Walking aids (Q2)	1.02 (0.13)	0-5	1-2	0	98.2
Locking (Q3)	2.11 (0.71)	0-5	1-4	0	18.2
Unstable (Q4)	1.82 (0.92)	0-25	1-4	0	47.3
Pain (Q5)	2.58 (0.98)	0-25	1-5	0	1.8
Swelling (Q6)	1.60 (0.95)	0-10	1-4	9.1	63.6
Climbing stairs (Q7)	1.71 (0.62)	0-10	1-3	9.1	38.2
Squatting (Q8)	2.11 (1.03)	0-5	1-4	12.7	34.5
Total score	74.69 (14.9)	0-100	39-95	0	0
Tegner Activity Scale					
Before injury	7.35 (1.91)	0-10	3-10	0	14.5
After injury	4.33 (2.7)	0-10	0-10	5.5	5.5

## Discussion

The most important findings of the present study were the good psychometric properties, good reliability, and good criterion-related validity of the Greek versions of the Lysholm and Tegner questionnaires. Our findings showed acceptable psychometric performance of these scores for Greek patients with various knee disorders. There were no difficulties in translating and culturally adapting the questionnaires, and the back-translation process corresponded very well with the original English versions.

The LKSS and TAS are two popular patient-reported instruments that have been used in numerous publications worldwide, and their reliability, validity, and responsiveness have been established for various knee problems [3,7-13]. So far, both questionnaires have been translated, validated, and culturally adapted into several different languages; including Italian [8], German [20], Spanish [16], Arabic [18], Turkish [17], and Chinese [14] for the LKSS; Iranian [19], German [21], and Chinese [15] for the TAS, and a Dutch version of both the LKSS and TAS [6]. These studies, involving 836 patients in total (ranging between 50-126 in each study), have shown good internal consistency, test-retest reliability, and external validity; their results are summarized in Table 5.

For the Gr-LKSS, we found an acceptable internal consistency (Cronbach’s alpha=0.779), which was consistent with the other studies, as seen in Table 5 (Cronbach’s alpha=0.60-0.91), indicating good overall homogeneity of the score, irrespective of our patient sample. Our sample population demonstrated various knee problems, including meniscus or cartilage lesions, multi-ligament injuries, and patellofemoral disorders; nevertheless, almost half of our population (26/55 patients) had at least an ACL injury (it was for them the LKSS was initially designed). Other studies of LKSS cultural adaptation have also used patient groups with various knee problems [8,17,18]. It is interesting that three studies with isolated ACL injuries have demonstrated a Cronbach’s alpha similar to ours (between 0.72 and 0.77) [14,16,20]; two studies with various knee problems have shown a Cronbach’s alpha between 0.68-0.90 [17,18], and one study including only patellofemoral disorders had a Cronbach’s alpha of 0.91 [8].

Gr-LKSS showed also excellent test-retest reliability (ICC=0.950, SEM=3.36, SDD=12.48%), which is consistent with the other studies, as shown in Table 5 (ICC=0.800-0.950) [6,8,14,16-18,20]. Re-administration of the test was performed within 48-72 hours in our study, which is comparable to the time range used in the other studies (three days to two weeks). To minimize the risk of short-term clinical change, no treatments were provided during this period. The difference in time should be large enough that respondents are not likely to remember or be influenced by their first set of responses when providing their second set, but small enough that genuine differences in scores are not likely to have occurred. The test-retest reliability was also excellent for both the Gr-TAS before injury (ICC=0.877) and after injury (ICC=0.876), which was also consistent with previous studies (ICC=0.710-0.990) [6,15,19,21]. The SEM and SDD were 0.66 and 24.79% for the Gr-TAS before injury and 0.96 and 62.55% for the Gr-TAS after injury respectively, which is considered logical, too, considering the variability of injuries across our sample. This means that two professional footballers can have similar Tegner scores before injury (9 or 10) but totally different scores according to their injury level [i.e. a simple meniscus lesion vs a multi-ligament injury with ACL, posterior cruciate ligament (PCL), and posterolateral corner (PLC)]. In the German TAS translation, Wirth et al. found a minimal detectable change of 1.4 points in 46 patients after ACL reconstruction [21], which is consistent with previous reports [3]. Huang et al. in their Chinese TAS translation found consistent SDD values in three groups of patients: healthy controls (0.43); two to three months after ACL (0.89); 3-12 months after ACL (0.44), but higher values in the pre-ACLR population (2.12), which may indicate low test-retest reliability [15]. According to Briggs et al., the SEM for patients with ACL injury was 0.64 with a resulting SDD of 1.77 [3]. The SEM and SDD in the study by Eshuis et al. (Dutch translation) were 0.40 points and 1.2 points respectively in 69 patients with ACL injuries [6], whereas in the study by Negahban et al. (Iranian translation), the SDD value was calculated to be 0.75 in 45 patients with ACL injuries [19]. In contrast, Swanenburg et al. in their validation study of LKSS and TAS in 52 patients after TKA found that the SDC of the Tegner-German was 2 points [12], which differs from the SDC of 1 point reported in ACL injury populations [3]. This is an indication that injury-specific scores like TAS may be less appropriate for older populations with knee OA.

Criterion-related validity was investigated by comparing the Gr-LKSS and Gr-TAS with three worldwide acceptable and validated questionnaires of knee function: IKDC, KOS-ADLS, and OKS. One of the main reasons for choosing these questionnaires was that all these have been translated and validated into Greek [26,28,30]. Similar studies of cultural adaptation (Table 5) have used IKDC [6,14,15], 36-Item Short Form Survey by Rand (SF-36)/RAND or SF-12 [6,14,16,17,19], Knee injury and Osteoarthritis Outcome Score (KOOS) [18,19], and also LKSS [21], Western Ontario and McMaster Universities Osteoarthritis Index (WOMAC) [14], Kujala’s score [17], Hip and Knee Questionnaire [16], one leg jump test [16], and healthy control groups [20,21]. Six studies have used more than one questionnaire for validation purposes [6,14,16,17,19,21].

Gr-LKSS yielded strong associations and highly statistically significant results with KOS-ADSL (r=0.713) and IKDC (r=0.735), indicating good criterion-related validity. For comparison, two studies that used IKDC for validation of LKSS yielded Pearson’s values of 0.60 [14] and 0.87 [6] both for patients having ACL injuries. However, the Gr-LKSS demonstrated weak associations with OKS (r=0.197), probably because OKS has been designed mainly for OA patients and our sample population included younger patients with various knee problems (other than OA). So far, only the Italian translation of the LKSS has shown strong associations with OKS. However, their 63-patient sample had a more homogenous and probably chronic knee problem (patellofemoral disorder), thereby possibly explaining the great discrepancy across our study. Additionally, compared to the questionnaires’ items for IKDC and KOS-ADL, OKS had more general type questions, possibly excluding more specific presentations.

Gr-TAS before-injury yielded poor associations with all three questionnaires. Values of Gr-TAS after injury showed statistically important associations with KOS-ADLS (r=0.635) and IKDC (r=0.675) but still weak correlation with OKS (r=-0.138). In the Dutch translation of TAS involving 96 patients with ACL injuries, where the IKDC form was used for validation, Eshuis et al. reported the r value as 0.42 [9]. Huang et al. in the Chinese translation of TAS also used IKDC form for validation in 78 patients with ACL injuries separated into four groups: healthy controls; two to three months after ACL; 3-12 months after ACL and pre-ACL population [15]. The overall r value was very good (r=0.79; p<0.05) but different among ACL groups and especially in contrast to normal controls. The group of ACL patients before receiving treatment had a value of 0.66 similar to our sample population. In this study, the TAS had also good correlation with Q1, 5, 7, 8 of the IKDC, whereas it showed poor correlation with Q6, 10 (r=0.00-0.53). This may imply that TAS has higher correlations with those subscales of IKDC that measure pain or level of activity than with those subscales that measure stiffness, swelling, locking, or catching. The other two studies of TAS, translations into Iranian [19] and German [21], had used KOOS and LKSS for validation in 100 and 56 patients with ACL injuries respectively and showed corresponding r values of 0.34 and a range of 0.60-0.77 accordingly.

As far as the floor and ceiling effects are concerned, the Gr-LKSS yielded no floor or ceiling effects, although several question-items (six out of eight) reported ceiling effects in more than 15% of the sample, thus scoring the worst possible score for these items. However, the fact that none reported a less than 39 (out of 100) total score ensures the elimination of ceiling effects. Regarding the Gr-TAS, ceiling effects were reported in 14.5% of the sample, scoring the highest score of 10 for the pre-injury administration of the Tegner. However, it is the nature of this single-item questionnaire that allows this high scoring, representing the maximum physical activity level of the patient; hence, we do not consider this score as a biased one.

Our study is not without limitations. Due to the study design, only preoperative data were collected and, therefore, the responsiveness of the Gr-LKSS and Gr-TAS were not assessed in the present study. Future studies should assess the sensitivity of these instruments to detect changes in activity levels over time following surgical or rehabilitative interventions. Another drawback is the small sample size; however, previous validation studies have used a similar number of individuals, and the sample size was large enough to reach statistical significance. Finally, the most critical limitation was the variability of knee disorders in our group, especially considering such a small patient sample. However, almost half of our patients had an ACL rupture, and both LKSS and TAS were initially developed to evaluate this type of injury. Gr-LKSS and Gr-TAS can be applied to Greek patients with different knee pathologies while their good criterion validity can strengthen the credibility of the other translated knee scores (Gr-IKDC and Gr-KOS-ADLS).

**Table 5:** Literature review of Lysholm and Tegner translation and validation studies LKS: Lysholm Knee Score; IKDC: International Knee Documentation Committee; OKS: Oxford Knee Score; SF-36: Short Form-36 questionnaire; KOOS: Knee injury and Osteoarthritis Outcome Score; SF-12: Short Form-12 questionnaire; HKQ: Hip and Knee Questionnaire; OLJT; one leg jump test; preop: preoperative; postop: postoperative; ACL: anterior cruciate ligament; TKA: total knee arthroplasty; NA: not applicable

Translated score(s)	Test-Retest Reliability (ICC)	Internal Consistency (Cronbach’s alpha)	Construct Validity (Pearson’s r)
Italian Lysholm (Cerciello et al., 2018) [8]: 63 patients with patellofemoral disorders (other scores: Kujala, Larsen, Fulkerson); validation: OKS	0.96	0.91	0.94
Arabic Lysholm (Ahmed et al., 2019) [18]: 100 patients with various knee problems (other scores: OKS, IKDC); validation: KOOS	0.8	0.9	0.70
Chinese Lysholm (Wang et al., 2016) [14]: 126 patients with ACL injuries (responsiveness: 6 months); validation: SF-36, IKDC, WOMAC	0.93	0.72	LKS / SF-36 (physical) = 0.70; LKS / IKDC = 0.60; LKS / WOMAC = 0.73
Turkish Lysholm (Celik et al., 2013) [17]: 70 patients with various knee problems; validation: Kujala knee score, SF-36	0.82	0.68	LKS / Kujala = 0.78; LKS / SF-36 = 0.61
Spanish Lysholm (Arroyo-Morales et al., 2019) [16]: 97 patients (42 for test-retest analysis) with ACL injuries; validation: SF-36, HKQ, OLJT	0.92	0.77	LKS / SF-36 = 0.50; LKS/ HKQ = -0.31; LKS / OLIT = 0.59
German Lysholm (Wirth et al., 2011) [20]: 50 patients with ACL injuries in 2 different groups: acute phase (n=12), late rehabilitation (n=16); validation: control group with no knee problems (n=22)	0.82 (acute phase), 0.84 (late phase)	0.73	Normal group = 96.8 ±5.1; acute group = 75.3 ±16.8; late group = 82.7 ±12.8
Chinese Tegner (Huang et al., 2016) [15]: 78 patients with ACL injuries (controls, preop, postop); validation: IKDC	0.71–0.99	NA	0.56–0.84
Iranian Tegner (Negahban et al., 2011) [19]: 100 patients with ACL injuries: 45 for re-test evaluation (other scores: Marx activity scale); validation: KOOS, SF-12 [Physical (P)-Mental (M)]	0.81	NA	Tegner / KOOS = 0.34; Tegner / SF-12 (P) = 0.29; Tegner / SF-12 (M) = 0.12
German Tegner (Wirth et al., 2013) [21]: 56 patients with ACL injuries in 2 different groups: acute postop phase (n=11), late postop phase (n=18); validation: healthy control group (n=20), LKS	Acute = 0.92; late = 0.97	ΝΑ	LKS acute = 0.77; LKS late = 0.60
Dutch Lysholm and Tegner (Eshuis et al., 2016) [6]: 96 patients (69 for test-retest analysis) with ACL injuries; validation: IKDC, RAND-36	0.93 (LKS); 0.97 (Tegner)	0.70 LKS part A; 0.83 LKS part B	LKS / IKDC = 0.87; LKS / RAND = 0.55; Tegner / IKDC = 0.42; Tegner / RAND = 0.48

## Conclusions

We believe this validation study is clinically relevant because it confirms that the Greek translated versions of LKSS and TAS are valid and comparable to the original versions and those translated into other languages. Both scores are short and easy to administer and interpret with only a minimal amount of time required for clinicians, patients, or researchers. The Greek translations and culturally adapted versions of Gr-LKSS and Gr-TAS are reliable and valid and can be used to assess the functional limitations of Greek patients with various knee disorders.

## Figures and Tables

**Figure 1:**
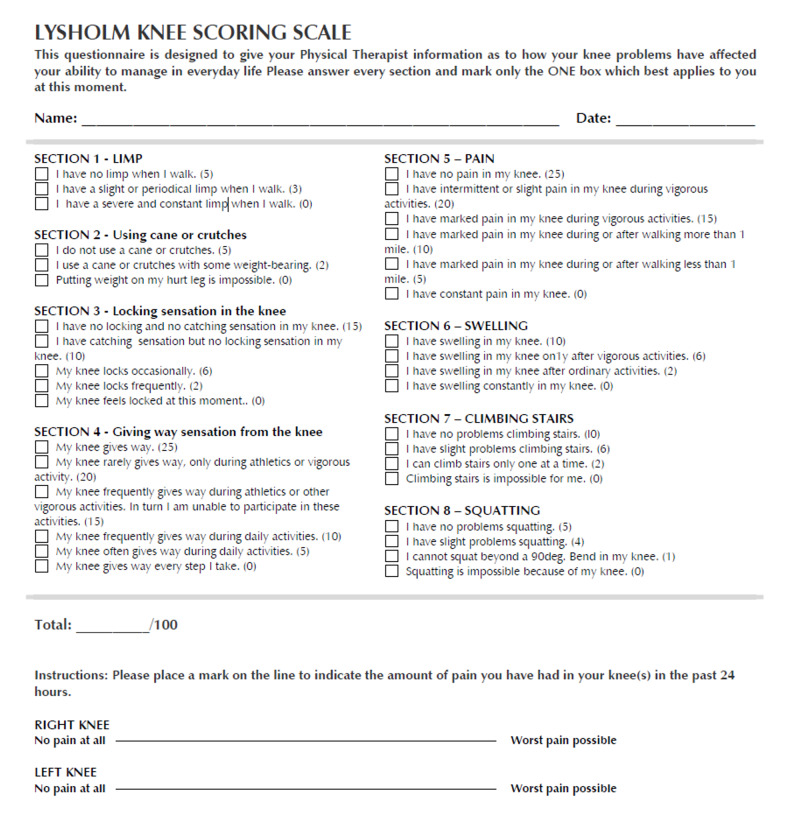
Lysholm Knee Scoring Scale

**Figure 2:**
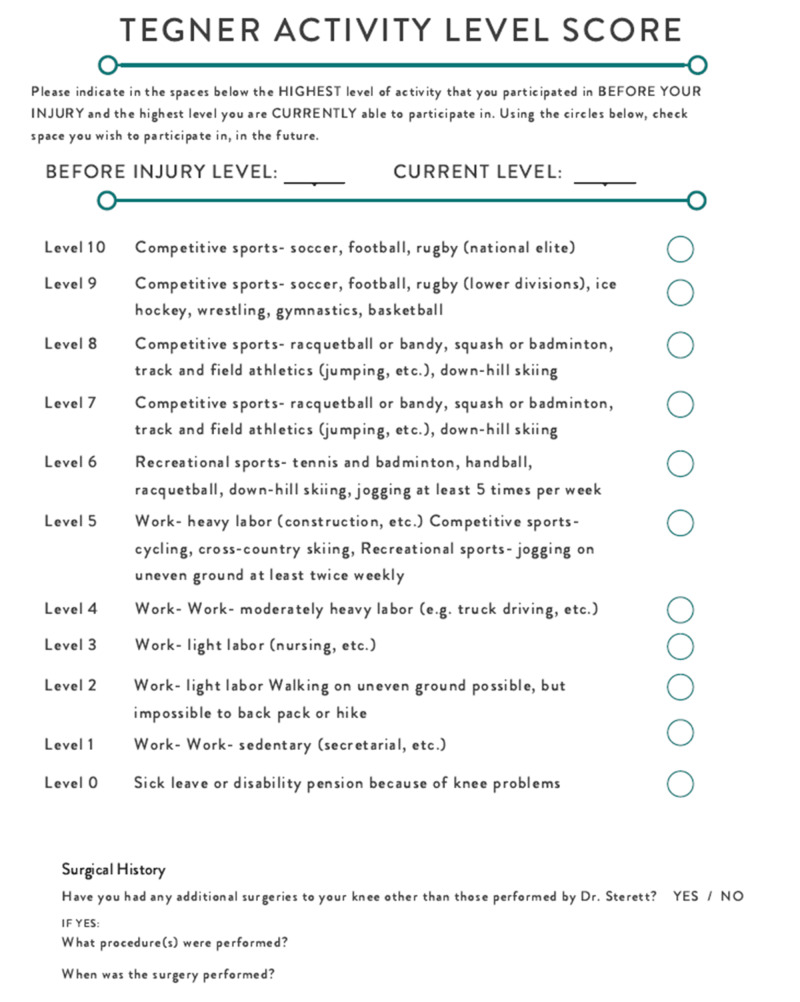
Tegner Activity Scale

## Appendices

APPENDIX 1

ΚΛΙΜΑΚΑ ΑΞΙΟΛΟΓΗΣΗΣ ΓΟΝΑΤΟΣ ΚΑΤΑ LYSHOLM

Οδηγίες: Παρακάτω παρατίθενται συνήθη συμπτώματα τα οποία παρουσιάζουν οι ασθενείς με προβλήματα στα γόνατα.

Παρακαλώ επιλέξτε την κατάσταση η οποία περιγράφει καλύτερα την περίπτωσή σας.

I.       ΧΩΛΟΤΗΤΑ (ΚΟΥΤΣΑΜΑΡΑ):

____ Δεν κουτσαίνω όταν περπατώ (5)

____ Κουτσαίνω ελαφρώς ή κατά διαστήματα όταν περπατώ (3)

____ Κουτσαίνω σοβαρά και συνεχώς όταν περπατώ (0)

ΙΙ.      ΧΡΗΣΙΜΟΠΟΙΗΣΗ ΜΠΑΣΤΟΥΝΙΟΥ Ή ΠΑΤΕΡΙΤΣΑΣ:

____ Δεν χρησιμοποιώ μπαστούνι ή πατερίτσες (5)

____ Χρησιμοποιώ μπαστούνι ή πατερίτσες με μερική φόρτιση (ελαφρό πάτημα) του ποδιού (2)

____ Η φόρτιση (το πάτημα) του τραυματισμένου ποδιού είναι αδύνατη (0)

III.     ΑΙΣΘΗΣΗ «ΚΛΕΙΔΩΜΑΤΟΣ» (ΜΑΓΚΩΜΑΤΟΣ) ΤΟΥ ΓΟΝΑΤΟΣ:

____ Δεν έχω αίσθηση «κλειδώματος» ή πιασίματος του γόνατος (15)

____ Έχω αίσθηση πιασίματος αλλά όχι «κλειδώματος» του γόνατος (10)

____ Το γόνατό μου «κλειδώνει» περιστασιακά (6)

____ Το γόνατό μου «κλειδώνει συχνά» (2)

____ Αισθάνομαι το γόνατό μου «κλειδωμένο» αυτή τη στιγμή (0)

IV.     ΑΙΣΘΗΣΗ ΟΤΙ ΤΟ ΓΟΝΑΤΟ ΜΟΥ «ΦΕΥΓΕΙ» (ΕΙΝΑΙ ΑΣΤΑΘΕΣ):

____ Το γόνατό μου δεν ποτέ ασταθές (25)

____ Μόνο κατά την άθληση ή άλλες δύσκολες δραστηριότητες (20)

____ Συχνά κατά την άθληση ή άλλες δύσκολες δραστηριότητες, «και δεν μπορώ να συμμετάσχω» (15)

____ Το γόνατό μου είναι συχνά ασταθές κατά τη διάρκεια καθημερινών δραστηριοτήτων (5)

____ Το γόνατό μου είναι ασταθές σε κάθε βήμα που κάνω (0)

V.      ΠΟΝΟΣ:

____ Δεν έχω πόνο στο γόνατό (25)

____ Έχω περιστασιακό  ή ελαφρύ πόνο στο γόνατο κατά τη διάρκεια έντονης δραστηριότητας (20)

____ Έχω σοβαρό πόνο στο γόνατό κατά τη διάρκεια έντονης δραστηριότητας (15)

____ Έχω σοβαρό πόνο στο γόνατό κατά τη διάρκεια ή μετά από περπάτημα πάνω από 2 Km (10)

____ Έχω σοβαρό πόνο στο γόνατό κατά τη διάρκεια ή μετά από περπάτημα λιγότερο από 2 Km (5)

____ Έχω συνεχή πόνο στο γόνατό (0)

VI.     ΠΡΗΞΙΜΟ:

____ Το γόνατό μου δεν είναι πρησμένο (10)

____ Έχω πρήξιμο στο γόνατό μόνο μετά από έντονη δραστηριότητα (6)

____ Έχω πρήξιμο στο γόνατό μετά από συνήθεις δραστηριότητες (2)

____ Έχω πρήξιμο στο γόνατό συνεχώς (0)

VII.    ΑΝΕΒΑΣΜΑ ΣΚΑΛΟΠΑΤΙΩΝ:

____ Δεν έχω πρόβλημα στο ανέβασμα σκαλοπατιών (10)

____ Έχω μικρό πρόβλημα στο ανέβασμα σκαλοπατιών (6)

____ Μπορώ να ανέβω μόνο ένα σκαλοπάτι τη φορά (2)

____ Το ανέβασμα σκάλας μου είναι αδύνατο (0)

VIII.   ΒΑΘΥ ΚΑΘΙΣΜΑ:

____ Δεν έχω πρόβλημα στο βαθύ κάθισμα (5)

____ Έχω ελαφρύ πρόβλημα στο βαθύ κάθισμα (4)

____ Δεν μπορώ να κάνω βαθύ κάθισμα με κάμψη του γόνατος μεγαλύτερη των 90^ο^ (2)

____ Το βαθύ κάθισμα είναι αδύνατο λόγω του γόνατός μου (0)

APPENDIX 2

ΚΛΙΜΑΚΑ ΔΡΑΣΤΗΡΙΟΤΗΤΑΣ κατά TEGNER

Παρακαλώ συμπληρώστε το μέγιστο επίπεδο δραστηριότητας σας πριν την κάκωση καθώς και το μέγιστο επίπεδο δραστηριότητάς σας τη στιγμή της εξέτασης

Πριν την κάκωση:   επίπεδο__________          Σήμερα:     επίπεδο___________

Επίπεδο 10 επαγγελματικός αθλητισμός - ποδόσφαιρο, ράγκμπι (επίπεδο εθνικής ομάδας)

Επίπεδο 9  επαγγελματικός αθλητισμός - ποδόσφαιρο (τοπικού πρωταθλήματος), πάλη, ρυθμική γυμναστική, μπάσκετ

Επίπεδο 8  επαγγελματικός αθλητισμός - τοιχοσφαίριση (squash), αντιπτέριση (badminton), στίβος (πχ άλμα εις ύψος), σκι (κατάβαση πλαγιάς)

Επίπεδο 7  επαγγελματικός αθλητισμός - τένις , τρέξιμο, motocross, χάντμπολ ή ερασιτεχνικά αθλήματα- ποδόσφαιρο, ράγκμπι, μπάσκετ, αντιπτέριση, τρέξιμο.

Επίπεδο 6  Ερασιτεχνικός αθλητισμός - τένις & αντιπτέριση, χειροσφαίριση (handball), σκι, τζόκινγκ (ελάχιστο 5 φορές/εβδομάδα)

Επίπεδο 5 Βαριά επαγγέλματα (οικοδομή κλπ.) Ανταγωνιστικά αθλήματα- ποδηλασία, σκι, ερασιτεχνικά αθλήματα- τζόκινγκ  σε ανώμαλο έδαφος (τουλάχιστον 2 φορές την εβδομάδα)

Επίπεδο 4 Μεσαίας βαρύτητας επαγγέλματα (π.χ. οδήγηση νταλίκας)

Επίπεδο 3 Ελαφρά επαγγέλματα (π.χ. νοσηλεύτρια)

Επίπεδο 2  Ελαφρά επαγγέλματα, βάδιση σε ανώμαλο έδαφος εφικτή αλλά όχι πεζοπορία στα βουνά ή μεταφορά σακιδίου πλάτης

Επίπεδο 1 Δουλειά γραφείου (π.χ. γραμματέας)

Επίπεδο 0 Αναρρωτική άδεια ή σύνταξη λόγω προβλημάτων στα γόνατα

Χειρουργικό ιστορικό γόνατος

Είχατε υποβληθεί σε χειρουργική επέμβαση στο γόνατο;?      Ναι   όχι

Εάν ναι: Τι χειρουργική επέμβση κάνατε?

Πότε έγινε η χειρουργική επέμβαση?

                                                                                                                                           ΣΥΝΟΛΟ_____/100

APPENDIX 3

APPENDIX 4
